# Young people’s data protection and privacy rights must not be neglected in the digital transformation of health: Insights, perspectives, and recommendations for the African context

**DOI:** 10.1371/journal.pdig.0000872

**Published:** 2025-06-05

**Authors:** Victor Oluwafemi Femi-Lawal, Temilola Aderemi

**Affiliations:** College of Medicine, University of Ibadan, Ibadan, Oyo State, Nigeria; McGill University, CANADA

The impact of digital transformation is rapidly spreading across healthcare. Today, providers are constantly driven by the need to improve patient outcomes by incorporating advancements in analytics, health data records, and AI. Digital health has the potential to revolutionize healthcare in Africa, solving problems of limited human resources, high disease burdens, and limited access [1].

The digital transformation of health increases opportunities for improved care, as well as potential risks from unethical use [2]. Children and youth, particularly those under the age of 17, are potentially more vulnerable to the harms posed by non-ethical data use and privacy infringements online [3]. Typically, young people below 17 lack the essential maturity needed to discern harm from good, making them vulnerable to harmful data extraction, human rights violation, privacy infringements, and other dangers online [4]. Additionally, incidences of data breach of vulnerable groups in the past reveal an urgent need to invest in data protection in healthcare [5].

However, in low-income contexts, the adoption of data protection regulations for digital health platforms is slow, with the majority of the population uninformed about these protections where they exist [6]. As a result of poor data governance frameworks and limited enforcement, many mobile health applications fail to satisfy privacy, safety, and ethical standards [2]. With these tools having access to confidential information, such security risks expose data to potential breaches. This lack of governance disproportionately affects young people [3].

Global frameworks such as the European Union’s Global Data Protection Regulation, the US’s Health Insurance Portability and Accountability Act (HIPAA), and the WHO Global Strategy on Digital Health 2020–2025 specify standards for health information privacy and security requirements in higher-income countries. These regulations spell out key considerations for young people under 17 years (such as data minimization, autonomy, confidentiality, and data protection impact assessment) which can potentially be adapted for African contexts, which face various challenges in protecting their health data.

A recent assessment of the digital health strategies of 10 African countries by Holly and colleagues showed a lack of specific considerations for young people. Among these countries, only one mentioned the right to privacy [3]. The evidence, therefore, strongly hints that Africa faces unique challenges in consumer protection and privacy, including weak regulatory frameworks, low digital literacy, increased vulnerability to cybersecurity risks, and low levels of youth inclusion in policy development [2]. Identified regulatory limitations include the lack of youth input, broad and unclear principles, conflicting values, and poor enforcement [7].

Despite these challenges, several African countries demonstrate a desire to prioritize young people’s rights. South Africa’s *Protection of Personal Information Act* (2020), for example, provides clear regulations for handling children’s data, requiring consent from competent persons, data minimization, and robust safeguards. Similarly, Kenya’s *Data Protection Act* (2019) mandates that health data be handled by professionals bound to secrecy and incorporates age verification and consent requirements. At least 29 other African countries have documented data protection policies, indicating growing recognition of personal data security needs [8]. Efforts are not limited to individual territories. Regional bodies like the African Union, Economic Community of West African States (ECOWAS), and Southern African Development Community (SADC) have established minimum data processing standards, although these often lack specific protections for young people [9].

Noting the challenges that countries and international communities face in protecting young people, there is a need for action to bridge existing gaps. For one, there is a need to devise inclusive data governance frameworks for accessing vulnerable groups’ data. As the digital health landscape evolves, significant thought must be given to designing effective mechanisms for recognizing, characterizing, and mitigating the peculiar risks faced by young people, such as non-consensual data sharing and exploitation. Policies must emphasize principles such as data minimization, age-appropriate consent, and data security [10]. From new ways to recognize young people’s digital footprints to more robust systems for age verification, there must be significant emphasis on the importance of designing and regulating these technologies to protect them.

While many vulnerable groups are aware their data is being digitized, few fully grasp the extent of this data use [11]. This indicates a need for training digital users on the negative impacts of improper technology use, engaging digital health experts in policy design, and full enforcement of data protection laws. Indeed, exploratory studies have suggested that educational interventions have a positive effect on knowledge and personal practices to ensure digital safety [12]. There is, therefore, a need for large-scale awareness and educational campaigns to draw public attention and drive advocacy.

Engaging young people in policymaking is important for inclusivity and effectiveness [13]. Advisory groups, networks, and committees such as Meta’s ‘Safety Advisory Council’ and the Africa CDC’s Youth in Digital Health Network (YiDHN) are examples of models that can be adapted by stakeholders. Such frameworks should address key barriers that typically emerge in attempts at youth engagement, such as social exclusion [14]. Additionally, such efforts should be accompanied by clear roadmaps for integrating recommendations into policy and practice [15]. Youth representation should also be ensured at decision-making boards and meetings.

Beyond regulations, digital literacy is crucial for empowering young people to navigate digital health safely. As Carah and colleagues (2024) highlight, keeping young people away from the digital world will only expose them to more harm long-term [15]. Therefore, governments, private companies, and other actors must invest more into digital literacy and capacity-building, educating young people on digital safety.

Collaborating with international bodies such as the ECOWAS, AU, and SADC, countries should adopt robust policies for handling young persons’ data across borders. These provisions should fully address key essentials, including security, interoperability, ethical use of AI, patient privacy, and transparency [8]. Recommendations outlined in the WHO’s Global Digital Health Strategy will be instrumental in these provisions, as countries create regulations and continually enhance them to meet people’s needs [10].

Safeguarding young people’s privacy and consumer rights is essential for ethical and effective digital health transformation in Africa. Ongoing dialogue and collaboration among stakeholders—young people, policymakers, healthcare providers, and digital innovators—are critical. By proactively addressing these concerns, Africa can leverage digital technologies to improve health outcomes while protecting the rights and well-being of its youth.

## References

[1] Victor AA, Frank LJ, Makubalo LE, Kalu AA, Impouma B. Digital health in the African region should be integral to the health system’s strengthening. Mayo Clin Proc Digit Health. 2023;1(3):425–34. doi: 10.1016/j.mcpdig.2023.06.003 37744952 PMC10512197

[2] Huckvale K, Prieto J, Tilney M, Benghozi P, Car J. Unaddressed privacy risks in accredited health and wellness apps: a cross-sectional systematic assessment. BMC Med. 2015;13:214. 26404673 10.1186/s12916-015-0444-y PMC4582624

[3] Holly L, Smith R, Ndili N, Franz C, Stevens E. A review of digital health strategies in 10 countries with young populations: do they serve the health and wellbeing of children and youth in a digital age? Front Digit Health. 2022;4:817810. 35373182 10.3389/fdgth.2022.817810 PMC8967994

[4] Holly L. Health in the digital age: where do children’s rights fit in? Health Hum Rights. 2020;22(2):49–54. 33390692 PMC7762891

[5] Seh A, Zarour M, Alenezi M, Sarkar A, Agrawal A, Kumar R, et al. Healthcare data breaches: insights and implications. Healthcare. 2020;8(2):133. 32414183 10.3390/healthcare8020133 PMC7349636

[6] Vayena E, Dzenowagis J, Brownstein J, Sheikh A. Policy implications of big data in the health sector. Bull World Health Organ. 2018;96(1):66–8. 29403102 10.2471/BLT.17.197426 PMC5791870

[7] Ferretti A, Adjei KK, Ali J, Atuire C, Ayuk BT, Banougnin BH, et al. Digital tools for youth health promotion: principles, policies and practices in sub-Saharan Africa. Health Promot Int. 2024;39(2):daae030. doi: 10.1093/heapro/daae030 38558241 PMC10983781

[8] Munung NS, Staunton C, Mazibuko O, Wall PJ, Wonkam A. Data protection legislation in Africa and pathways for enhancing compliance in big data health research. Health Res Policy Syst. 2024;22(1):145. doi: 10.1186/s12961-024-01230-7 39407232 PMC11479556

[9] Greenleaf G, Cottier B. International and regional commitments in African data privacy laws: a comparative analysis. Comput Law Secur Rev. 2022;44:105638.

[10] World Health Organization. Global strategy on digital health 2020—2025 [Internet]. World Health Organization; 2021 [cited 2025 Jan 27]. Available from: https://www.who.int/docs/default-source/documents/gs4dhdaa2a9f352b0445bafbc79ca799dce4d.pdf

[11] Nguyen T, Yeates G, Ly T, Albalawi U. A study on exploring the level of awareness of privacy concerns and risks. Appl Sci. 2023;13(24):13237.

[12] Albrechtsen E, Hovden J. Improving information security awareness and behaviour through dialogue, participation and collective reflection: an intervention study. Comput Secur. 2010;29(4):432–45.

[13] Layton D, Forst M, Broderick B. A Rights-based approach to youth inclusion in public health policy. Am J Public Health. 2022;112(8):1120–2. doi: 10.2105/AJPH.2022.306944 35709428 PMC9342805

[14] Okpalauwaekwe U, Ballantyne C, Tunison S, Ramsden VR. Enhancing health and wellness by, for and with Indigenous youth in Canada: a scoping review. BMC Public Health. 2022;22(1):1630. doi: 10.1186/s12889-022-14047-2 36038858 PMC9422134

[15] Carah N, Demaio S, Holly L, Kickbusch I, Williams C. Beyond banning: our digital world must be made safer for young people. Health Promot J Austr. 2025;36(1):e938. doi: 10.1002/hpja.938 39694686

